# Intelligence and Cognitive Development: Three Sides of the Same Coin

**DOI:** 10.3390/jintelligence5020014

**Published:** 2017-04-13

**Authors:** Anik de Ribaupierre, Thierry Lecerf

**Affiliations:** Faculty of Psychology and Educational Sciences, University of Geneva, 40 Bd du Pont d’Arve, 1205 Geneva, Switzerland; Thierry.Lecerf@unige.ch

**Keywords:** intelligence, cognitive development, individual differences, working memory, processing capacity, attentional control, inhibition

## Abstract

Research on intelligence, mainly based on correlational and factor-analytical work, research on cognitive development, and research in cognitive psychology are not to be opposed as has traditionally been the case, but are pursuing the same goal, that is, understand how the human being adapts to his/her own, complex environment. Each tradition of research has been focusing on one source of variation, namely situational differences for cognitive psychology, individual differences for psychometrics, and age differences for developmental psychology, while usually neglecting the two other sources of variation. The present paper compares those trends of research with respect to the constructs of fluid intelligence, working memory, processing speed, inhibition, and executive schemes. Two studies are very briefly presented to support the suggestion that tasks issued from these three traditions are very similar, if not identical, and that theoretical issues are also similar. We conclude in arguing that a unified vision is possible, provided one is (a) interested in the underlying processes and not only in the experimental variations of conditions; (b) willing to adopt a multidimensional view according to which few general mechanisms are at work, such as working memory or processing capacity, inhibition, and executive schemes; and (c) granting a fundamental role to individual differences.

## 1. Introduction

This paper, like this entire special issue, is addressing the commonalities and the divergences between three trends^1^ concerning the study of intelligence, which have been traditionally opposed, in psychology, even if often implicitly: Psychometrics, cognitive psychology, and developmental psychology. These three traditions are considered to differ widely, have largely ignored or criticized one another and have followed different routes in their development despite recurrent calls for their rapprochement [1,2,3,4,5]. Yet, all pursue the same objectives, namely attempt to understand the behavior observed in tasks assessing reasoning, memory, or in other complex tasks, and more generally how the human being adapts to his/her own, complex environment. They all tend to focus on only one (rarely two) aspect of the complex interactions between human beings and their environment. However, Cattell [6,7] advocated very early the use of multivariate research, and proposed the data box, that schematizes the study of individuals, variables, and occasions (which can also be understood as long term occasions, i.e., development). 

The study of intelligence is often, although not always, understood as the psychometric approach of individual differences by means of standardized tests tapping cognitive or intellectual abilities^2^. It has also been based on correlations between performance in cognitive tests, focusing on the existence of a second-order factor (i.e., a general intelligence factor) and first-order factors (i.e., group or broad abilities factors). Therefore, it is frequently the case that several tasks are administered to the same individuals (multivariate approach) unless using a task that has been validated as reflecting rather reliably the general factor (e.g., the Raven Progressive Matrices, prototypical task of fluid intelligence). Psychometricians interested in intelligence have very rarely adopted a developmental perspective, whether in childhood or across the lifespan, even though the initial works on intelligence were based on children; age differences were used to assess the concept of mental age or intellectual level, based on differences between children’s behavior in various short tasks [8]. Hunt nicely paraphrased, in his broad review of human intelligence, the almost exclusive focus on young adults as follows: “*All sophomores are humans. Not all humans are sophomores*” ([9], p. 171). 

Experimental psychology of cognition or cognitive psychology, as it has now been labeled for decades, has almost exclusively focused on variations in situational differences or experimental manipulations (between-treatments) and their effects on human beings, and interpreted their effects in terms of underlying processes. We will use the term “situations” (or “situational differences”) throughout this paper, to refer to the variable side of Cattell’s data box [7], contrasting it to the two other points of view to take into consideration, i.e., individuals and occasions (see also below). The term “variable” seems somewhat ambiguous in the present context, as it also refers to variability. The term “experimental manipulation” is too narrow, as a focus on situations might also include methods not necessarily restricted to experimentation in the strict sense (for instance, observations, use of tests, etc.). Incidentally, Cattell also used the term “situations”. Cognitive psychology uses experimental laboratory tasks, not standardized; it focuses essentially on the situation and its variation created experimentally, and searches for underlying processes. Hunt’s quotation (above) can also be applied to cognitive psychology. In both the psychometric and the cognitive approaches, developmental differences are merely considered as a special case of individual differences, themselves largely ignored. It is as if the processes observed in adults were all already present in children or only had to unfold. Yet, like Piaget mentioned, a cognitive function cannot be fully understood if its genesis (development) is not also studied.

Developmental psychology also uses essentially experimental, unstandardized tasks, and focuses on age differences in various tasks, whether in childhood or across the lifespan. It has been forgotten that Piaget himself used the term of intelligence, for instance in his major, relatively early work on the “Origins of Intelligence” or on the “Psychology of Intelligence” [10,11] that established the basic principles of his theory as it applies to psychology. Lautrey and de Ribaupierre [12] remarked that, in the Francophone tradition, the study of intelligence was not restricted to the use of standardized tests of intelligence nor to the study of individual differences, but also concerned developmental studies of cognition. This is only recently that researchers working in the tradition of Piaget (or of followers) abandoned the term of intelligence to replace it by that of cognition. Piaget and his co-workers (e.g., [11]) had themselves a very critical look at the differentialist perspective on intelligence, considering that it was totally atheoretical and focused only on scores and performance, that is, on products rather than on processes. In contrast, Piaget advocated to pursue the study of underlying mechanisms, like much of today cognitive psychology. In contrast with cognitivists, however, he searched for general mechanisms such as adaptation, equilibration, etc., rather than for specific processes. Yet, like cognitivists, Piagetians and more generally child developmental researchers were not interested in individual differences, relegating them to issues of applications. A further specificity of developmental psychology, in particular Piagetian, is to have looked for qualitative differences, in contrast with the psychometric approach and a good part of the cognitive psychology, which have always been quantitative. 

Our argument, in the present paper, will be that the three approaches are complementary, and should be adopted simultaneously if we ever want to understand cognition. We are of course neither the first, nor the only ones to plea for such a rapprochement ([1,2,4,5,13,14,15]; see also the present special issue).

## 2. Initial Suggestions of Bridging

A number of suggestions to bridge these approaches remained without response for a long time, even though their integration is obviously needed if one wants to understand the diversity of psychological data which have been collected so far [5]. The focus on situations has led to a decomposition into extremely fine processes and an almost total neglect of potential more general mechanisms. In contrast, looking across individuals (differential psychology) or looking across ages (developmental psychology) has led to a search for general processes or mechanisms at the expense of finer analyses of situations. 

Among the major precursors, Cronbach [2] provided profound observations on the growing divergence of “the two disciplines of scientific psychology” (experimental versus correlational or differential psychology), each of which was interested in what the other one neglected, that is, between-treatments variance (treatment variation) and variance among organisms (individual differences), respectively. According to Cronbach ([2], p. 671), the correlational psychology (the correlators) “*asks a distinctive type of question and has technical methods of examining whether the question has been properly put and the data properly interpreted*”. He suggested that differentialists (correlationists) and experimentalists be inspired by each other, and proposed for instance to the differentialists to introduce construct validation into test theory and to the experimentalists to adopt multivariate approaches. From a methodological point of view, he called for the “joint application of experimental and correlation methods” (p. 679). From a theoretical point of view, Cronbach suggested that both experimentalists and differentialists could test the same theoretical “hidden constructs” [16]. In sum, Cronbach [2] suggested that experimentalists and differentialists should have “a common theory, a common method, and common recommendations” (p. 683). We fully agree with Cronbach: Integration should be done at both a methodological and a theoretical level. Nevertheless, we consider that differentialists also addressed specific theoretical issues, like the assessment of the relationships between several observed or latent variables, which form a system [17]. Thus, the differentialists were more interested by the structure. Moreover, Cronbach did not address the developmental perspective. 

Cronbach’s call, although frequently acknowledged, remained relatively ignored until recently. In France, Reuchlin [4,18] was more successful. He asked that both general psychology and differential psychology be forced to account for the phenomena described and the method used by the other discipline: Generalists should be able to incorporate individual differences in their theories, at the risk, otherwise, to develop theories applicable to only a very limited subset of human beings; differentialists would be well inspired to also resort to experimental methods in order to better understand the processes involved in their tasks. Reuchlin [4] also offered a few more precise suggestions to bridge differential psychology and Piagetian theory: Piaget’s concept of equilibration might be used to feed some theory into the general factor identified by differentialists, while Piaget’s concept of “horizontal decalage” (e.g., time delay in the acquisition of concepts supposed to be of the same complexity, i.e., belong to the same structure) could find some explanation in the group factors of the differentialists by interpreting these decalages in terms of individual differences. Reuchlin’s suggestions were indeed followed by a number of Francophone researchers [19,20,21] leading to a tradition that Larivée and collaborators qualified as the “French connection” [22].

For the remainder of this paper, we will focus on only a few instances of rapprochement, in particular on attempts to bridge the psychometric concept of fluid intelligence (*Gf*) with cognitive studies on Working Memory (WM) capacity, inhibition, or processing speed on the one hand, and with developmental studies as exemplified by neo-Piagetian approaches on the other hand. Although several basic mechanisms could be causally responsible for knowledge acquisition, we focus in this paper on WM, processing speed, and inhibition, because these mechanisms could be germane to explain both individual and developmental differences, and hence could allow unifying the three approaches under a common theory^3^. Because the concepts used by the cognitivist approach are also used by the developmental tradition, this attests once again that these three traditions attempt to understand the same phenomena, and reach similar interpretations, even though from a different standpoint [13]. To illustrate how tasks issued from the three traditions do indeed interact with one another, and that these three approaches address similar theoretical issues, we will very briefly present some empirical data from our lab. We will then conclude in highlighting some processes that seem to be common to the three approaches, while arguing for the necessity to consider a small set of general mechanisms, rather than a single global factor, at work in many situations, present in most age groups, but differing in weight depending on situations as well as on individual and developmental differences.

## 3. Fluid Intelligence and Cognitive Psychology

Psychometric intelligence research, in addition to having thoroughly studied individual differences and provided a number of multivariate methodologies, has firmly established the distinction between fluid and crystallized intelligence (*Gf* and *Gc*) [23]. Although both are considered as correlated general factors underlying a large number of tasks, crystallized intelligence rests on accumulated knowledge and experience (general knowledge, vocabulary), while fluid intelligence is the capacity to reason and solve novel problems and was considered as relatively independent from past knowledge. The former is considered to be the product of education and culture, while the latter is believed to be closer to the biological underpinnings of behavior. These two factors were considered as two general mechanisms by Baltes, and were respectively labeled pragmatics and mechanics of cognition (e.g., [24]). 

These two broad factors reflect the fact that a large number of tasks tend to class individuals in a relatively similar manner, that is, interindividual differences are more similar among fluid intelligence tasks on the one hand, and among crystallized intelligence tasks, on the other hand, than across tasks. A general factor or a group factor is nothing more than a psychometric latent variable, and does not necessarily correspond to a given process or a class of processes. In simulation studies, van der Maas and collaborators have elegantly demonstrated that a general factor can be obtained from a collection of totally independent processes [25]. Thus, observing a general factor does not imply that there is a single general underlying mechanism. Moreover, the *g* factor still requires a psychological interpretation. 

The Cattell-Horn’s distinction between fluid and crystallized intelligence was later supported by the “Three-stratum” theory proposed by Carroll [26]. This model is a taxonomy, based on more than 460 human cognitive ability datasets, and has become largely consensual nowadays when fused with the Cattell-Horn model (CHC model—see [27]). It suggests three levels of group factors: Narrow abilities (Stratum I), which are included in Broad abilities (Stratum II), which are in turn correlated and grouped into a general factor (Stratum III). Some of these broad abilities correspond closely to the three constructs mentioned above. Within the Stratum II abilities, Carroll proposed indeed the existence of a broad general memory and learning ability (Gy), which includes WM (the CHC model includes a short-term working memory broad ability Gwm). As concerns speed of processing, Carroll distinguished a broad processing speed ability (Gt, defined as a Reaction and decision time) and a broad cognitive speediness ability (Gs). There is, to our knowledge, no specific Inhibition group factor. However, there have been very few psychometric studies, or tests, of inhibition. Yet, coming from a different perspective, Miyake and collaborators conducted a number of studies on individual differences in executive functions and inhibition, using confirmatory factor analysis [28,29,30]. They showed that, within the constructs often considered to reflect inhibition, Prepotent response inhibition and Resistance to distractor interference were both related but independent from Resistance to Proactive Interference (PI). 

As mentioned above, most cognitivists shied away from looking for global mechanisms. Yet, in the last twenty years or so, a few mechanisms have been considered to be causally responsible for knowledge acquisition, and hence for intelligence. First, it has been suggested that intelligence might be based on speed of information processing [31,32,33,34]. Working Memory (WM) has frequently been suggested to be one of the constructs widely used in cognitive psychology that might be most closely related to the general factor (*g*-factor), and more particularly to *gf* [35,36]. For instance, Kyllonen and Christal [37] were the first to assume that reasoning ability, as defined by psychometricians and obviously one instantiation of *gf*, is very close to WM capacity. More specifically, they suggested that individual differences in intelligence are due to at least four sources, namely processing speed, breadth of declarative knowledge, breadth of procedural knowledge, and working memory, the latter being the most important. Similarly, Engle and collaborators demonstrated the existence of a strong link between individual differences in WM as studied with complex reading span tasks and fluid intelligence [38,39,40]. Thus, for some time, WM has been presented as one of the main constructs, if not the only one, underlying fluid intelligence, and the overlap was considered to be very large. According to Gignac [41], fluid intelligence shares close to 60% of its variance with WM capacity. We do not have space here to discuss the results of years of research on WM led in different perspectives, and the various controversies. Let us just remind that, according to a general present consensus, WM serves to maintain temporarily and process information essentially for use in other cognitive tasks and that its capacity is very limited. For instance, a complex span task requires to retain words (or other information) to be retrieved later while processing other information (reading a sentence, performing an arithmetic operation). A most important characteristic, present in many working memory tasks, is the necessity to maintain the information when concurrent processing information, distraction and/or attentional shift is also taking place. For instance, complex span tasks require to perform an operation while retaining one word and retrieve all words at the end of a sequence. Likewise, the *n-back* task, very popular nowadays in the working memory field, combines memory for an item (consonants or digits or other simple information) with interfering information of the same type (sequence of consonants or of digits) that should be inhibited, thereby forcing a shift between activation and inhibition of information of the same type. Thus, rather than considering that WM itself is a combination of storage and processing, we suggest that the dual tasking present in many WM tasks serves to prevent the formation of larger informational chunks using grouping strategies between the elements of information to retain. This allows the experimenter to assess the WM capacity of an individual more reliably than by means of a simple span task in which items can be more easily grouped provided they are known by the individual. It demonstrates neither that WM is a process, nor that it explains fluid intelligence. We prefer to assume that WM and intelligence refer to a set of tasks that share the same processes. What is indeed common to *gf* and WM is the novel characteristic of the task, and the relative uselessness of previous knowledge (unlike crystallized intelligence tasks). Also, *gf* tasks are often complex and therefore require large processing capacities, which will be better measured by WM tasks. It should also be mentioned that Gignac and Watkins [42] showed that the association between WM capacity and *gf* is not special, but can be subsumed in the link with general intelligence; it became nonsignificant (− .10, *p* = .152) after the effect of general intelligence was controlled for.

More recently, Engle and collaborators [43] also showed the link of WM with executive attention, that is, with tasks requiring not only a certain amount of processing capacity but also an ability to disengage attention and to reengage it into another part of the stimulus. In this latter approach, Engle and collaborators are, actually, making suggestions that are very close to those defended by neo-Piagetians, in particular by Pascual-Leone, as it will be shown below. This proposal of the importance of executive attention seems rather similar to the one made previously by these authors regarding WM. 

Linking psychometric intelligence to WM tends to underline the fact that intelligence tasks require a relatively large processing capacity, rather than simply a large storage as Colom and collaborators argue [44]; describing a link between executive attention and *gf* tasks merely displaces the stress on an attentional component (engage and disengage attention) also present in *gf* tasks. Our argument is that both are needed in most complex tasks, as are also other processes. More generally and most importantly, one should make a clearer distinction between systems or processes on the one hand, and tasks on the other hand. Psychometricians and cognitivists have too often established a direct equivalence between tasks and processes, as if each task corresponded to a single, and different process. Tasks and processes are therefore confounded and considered equivalent. For instance, there are WM tasks, relatively well defined, but there is not necessarily a WM system. In contrast, we believe that most tasks are underpinned by several processes that are shared across tasks in a different dosage. This requires to conduct more detailed task analyses that focus on what the problem solver is doing (i.e., response processes). For instance, all tasks designed to tap executive attention, working memory, or executive functions require attentional control, in particular inhibition of irrelevant information, and this is the reason why they correlate. The same processes are also involved in fluid intelligence tasks, additionally to other aspects such as previous knowledge, logical rules, etc. However, the relative strength of each process might vary from task to task, explaining why the overlap is rarely total. 

## 4. Developmental Aspects

Neo-Piagetians developed their theories on the basis of, and sometimes against, the Piagetian theory, in order to link it with the “information processing approach”, that is, in fact with cognitive psychology. They retained a number of characteristics, notably a constructivist approach; most of them abandoned the definition of qualitative stages subtended by logical structures. In addition, neo-Piagetian theories attempted to better account for the diversity of situations and for learning effects [45,46,47,48]. Like Piaget, however, they also searched for general mechanisms, while granting much more attention than Piaget to situations. Among the neo-Piagetian perspectives, Pascual-Leone’s theory will be given more attention in this paper, not only because it is the first to have been proposed, but also because it is the most ambitious one by integrating all three approaches, developmental, cognitivist and differential [49,50]. A common characteristic of all neo-Piagetian models, in line with Pascual-Leone’s suggestions, is to propose that a general limit in processing resources sets constraints on the cognitive level that can be reached in any one task. Development consists in a slow increase of this limit, together with a development in strategies and knowledge. Those processing resources correspond to the construct of WM capacity discussed above, and have been termed somewhat diversely by the neo-Piagetians as mental capacity, attentional capacity, M-capacity, etc. 

More precisely, Pascual-Leone assumed that the increase of attentional capacity (i.e., M-capacity or M-power) during childhood is one of the causal factors of general cognitive development [51], see also [46,52,53]. Pascual-Leone distinguishes the “field of working memory or centration” and the “field of mental attention” [54]. The former contains the schemes or mental representations built by the individual that are more or less directly activated (i.e., effortlessly) by the stimulus [55] and roughly corresponds to what is commonly understood in the literature under the label “working memory” (e.g., [56]). The field of mental attention contains the schemes that need to be activated further by the M-operator, the I-operator, and executive schemes; note that this definition of attention is close to the focus of attention in Cowan’s model of working memory [57]. In Pascual-Leone’s model, M-capacity is probably the most important factor from a developmental perspective, although not the only one, because it imposes an upper limit on cognitive performance and increases regularly with age. This growth is essentially driven by biological mechanisms, but the newly developed capacity might require in turn some learning and more generally experience to be better exploited, in particular by developing executive schemes. Because of their more limited processing capacity, young children fail or adopt more elementary reasoning in some complex tasks than older children or adults, who are able to solve them. If a child has not yet reached the corresponding M-capacity level, he/she will fail and/or make typical errors. Elaborate task analyses are needed to determine the minimal complexity for a task to be solved, termed M-demand. M-capacity is considered as a necessary but not a sufficient condition to perform at a given level. This is why an implicative relation is assumed between the task’s difficulty and M-capacity rather than an equivalence one (correlational). It means that functioning at a certain level in a cognitive task requires a given M-capacity; but having developed a sufficient capacity does not guarantee that the task be passed, because M-capacity is not the only process on which the task draws. In particular, the child has to master the content of the task and to develop strategies; the task might also present misleading features that might need additional inhibitory processes. Indeed, Pascual-Leone [51,58] proposed that several other mechanisms like inhibition, executive schemes, etc. (operators I, F, L, etc.) are engaged, more or less strongly depending on the task. 

Other neo-Piagetian models were proposed, most of which stipulating that developmental stages rest on the growth of central processing resources [45,59,60,61]. They also retained from the Piagetian theory a qualitative description of substages, although profoundly modified, while attempting like Pascual-Leone to better account for the diversity of the situations. Note, incidentally, that a quantitative increase in processing capacity may lead to a qualitatively more complex way to solve a problem. The interest of Pascual-Leone’s theory, for the present paper, is to offer a wider generality because of its multidimensionality. The hypothesis of several underlying mechanisms makes possible not only refined task analyses, but also a combination of developmental and individual differences. All these mechanisms are supposed to be present in all individuals, but their influence and their interactions may vary [62]. For instance, Pascual-Leone showed that field dependent individuals are more prone to misleading features, for instance in Piagetian tasks, and may therefore require more M-capacity to counteract them, accounting for some delay in solving a number of tasks [62,63].

In sum, there are several convergent but also divergent points between neo-Piagetians and cognitivists. Like cognitivists, neo-Piagetians and more generally developmentalists call on a construct of processing resources, such as WM capacity or attentional capacity, inhibition, executive attention [16,64]. Note that lifespan developmentalists have also searched for general mechanisms, such as processing speed, or inhibition (e.g., [24]). The major difference with cognitivists is that developmentalists look for age differences in those processes and regard them as generalizable, to a certain extent, across tasks, addressing issues such as “how does WM develop”, and thereby explaining age differences in fluid intelligence, or in all sorts of cognitive tasks. Yet, they are also interested in how different situations may account for variations in cognitive functioning. Few developmentalists, however, integrate inter- and intraindividual differences in their model, except Pascual-Leone’s and, to some extent, Fischer’s model [61]. This interest for individual differences is, usually, restricted to correlations across tasks, which themselves rest on interindividual differences, but none describes different developmental trajectories for different types of children.

## 5. An Illustration of the Relationships between Fluid Intelligence, Working Memory and Inhibition

From a more empirical point of view, we want to argue not only that the three perspectives are similar in their objectives and assumptions, but also that tasks developed within those traditions, such as fluid intelligence tasks, working memory tasks, and Piagetian tasks tap similar processes. These tasks are generally novel for the individual and do not require much pre-established knowledge. They also present misleading or distracting characteristics that call for attentional control. They differ, however, in the extent to which they rely on additional processes and/or on previous experience and knowledge. No task ever calls for a single process, and the relative part of general mechanisms as contrasted with specific ones may vary, leading to large interindividual differences. Thus, correlations are very high, but the overlap is not total. 

**Figure 1:**
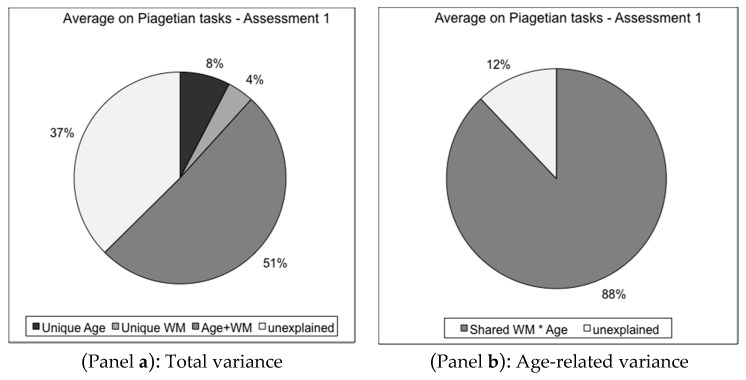
Regression and commonality analyses: Piagetian tasks. Assessment 1 for those tasks (Year 2 of the longitudinal study). Children aged 6–11 years, *N* = 100. Age and working memory tasks were used as predictors on each of the Piagetian tasks. Commonality analyses were then conducted to assess the variance due uniquely to each predictor and the shared variance. The figure displays the average value across the Piagetian tasks, for the total variance (Panel **a**) and for the age-related variance only (Panel **b**).

We conducted several studies in our lab, which combined tasks borrowed from more than one tradition. To illustrate some of the arguments raised in the present paper, we will summarize very briefly two of those studies, in which either WM tasks and Piagetian tasks on the one hand, or WM tasks and fluid intelligence ones on the other hand, were used simultaneously (for more details [65]). First, in a longitudinal study conducted with children aged 5 to 10 years at the onset of the study and examined each year for five years, we used WM tasks identified with the cognitivist trend or the neo-Piagetian trend, and Piagetian tasks. Three WM tasks, the CSVI task [49], the Peanut tasks [45,66], and three Piagetian tasks, the Balance task, the Islands task and the Line Foldings task [67,68,69] were administered together with other tasks, to 120 children on the second, third and fifth years of the study. The Piagetian tasks represented different domains: logical reasoning, representation of space and mental imagery, respectively. We were particularly interested in the extent to which WM tasks accounted for age differences in the Piagetian tasks. Following a method widely used by Salthouse [70], regression and commonality analyses were conducted. Age and WM performance were entered as predictors to estimate the part of variance in each of the Piagetian tasks accounted for by Age alone, WM alone (three tasks), and the interaction of Age and WM, respectively. Results showed that an important part of the total variance observed in the Piagetian task was accounted for by these two predictors and their interaction, ranging from 63% in the first assessment (year two of the study, children aged 6 to 11 years), to 43% on the second assessment (children aged 7 to 12 years), and 40% on the third assessment (children aged 9 to 14 years) when averaged across all Piagetian tasks. Interestingly, most of this variance was shared by Age and WM together, and the unique variance due to Age alone or to WM alone was very small. The decrease in the variance accounted for over the years reflects most probably an increase in the part accounted for by other causes than those linked to WM capacity, such as strategies and/or learning. Figure 1 reports these results for the first assessment. The same logic was followed to estimate the part of the age-related variance (Figure 1, Panel b), rather than the total variance (Figure 1, Panel a), in the Piagetian tasks, due to the WM tasks. It was close to 90% for each assessment (88%, 92%, and 86% for the first, the second, and the third assessment, respectively), which is extremely high. When the analysis was undergone the other way around, that is, when using Piagetian tasks to account for age-related variance in the WM tasks, the amount of variance explained was much lower. This suggests that WM is a better predictor of functioning in the Piagetian tasks than the reciprocal prediction. This is not surprising, as WM tasks involve probably a lesser amount of different processes and strategies than Piagetian tasks. Nevertheless, taken together one can conclude that these two types of tasks are very close. 

**Figure 2:**
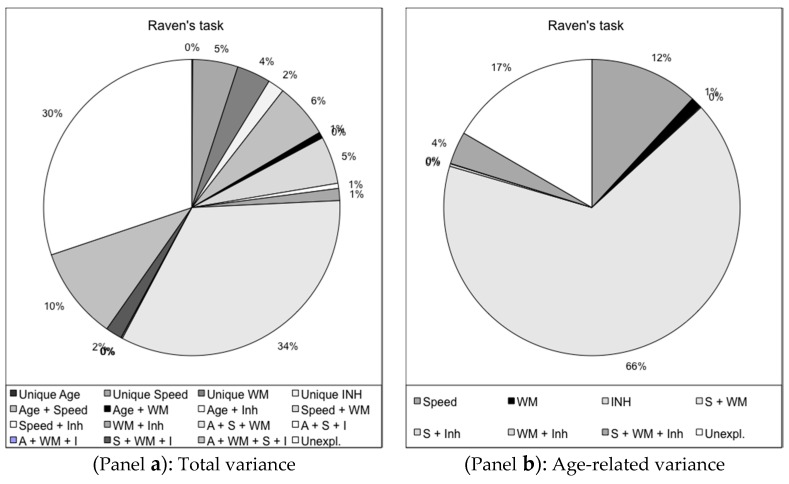
Regression and commonality analyses: Raven’s task (Children and young adults). Children aged 8–12 years and young adults (20–30 years of age), *N* = 262. Age, as well as working memory, processing speed and inhibition tasks were used as predictors on the Raven task. Commonality analyses were then conducted to assess the variance due uniquely to each of the four groups of predictors and to their shared variance (two by two, three by three and all four predictors). (Panel **a**): total variance. Only variance accounted by several predictors together was very important, in particular Age and Speed together (6%), Speed and Working Memory (WM) (5%), Age, Speed and WM (34%), all four groups (10%). (Panel **b**): Age-related variance: Speed and WM together explained about two thirds of the age-related variance (66%).

The second study to be provided as an illustration of the similarity between tasks issued from different traditions included WM tasks and a fluid intelligence task. It was a cross-sectional lifespan study, conducted with children, young adults, and older adults. A large multivariate design was used, including four groups of tasks: processing speed, inhibition, WM tasks, and the Raven’s Matrices (a fluid intelligence task). They were administered to approximately 530 individuals: children aged 8 to 12 years, young adults (20–30 years), and older adults aged 60 to 88 years. Two age groups were created: Young (children and young adults) and Adults (young and old adults). Like in the study previously described, regression and commonality analyses were used, with four sets of variables, Age, WM (two tasks), Inhibition (three tasks), and Processing speed (three tasks), as predictors of the performance in the Raven’s Matrices. The predictors were entered alone (e.g., Age, WM, etc.), in combination two by two (e.g., Age with WM or Age with Inhibition, etc.), or three by three and finally all together. To summarize very briefly, the total variance accounted for in the Raven’s was 70% in the Young group (see Figure 2), and 60% in the Adult group (not reported here). Most of this variance was explained by a combination of predictors, in particular Age, WM, and Speed, while the variance linked to a unique given predictor was very small, even null. This demonstrates that the Raven’s Matrices, like most other tasks, are underpinned by several processes. Once more, our interest was in the decomposition of the age-related variance; it represented about 51% of the total variance in the Young group, and 40% in the Adult group. The largest part of it was explained by a combination of Processing speed and WM in both groups, but more so in the Young group (66%, see Figure 2, Panel b); Inhibition played a very small role, but was slightly more important (four and five percent when associated with WM, and with WM and Speed, respectively) in the Adult group. 

In sum, both studies showed that the three types of tasks (Piagetian, WM and fluid intelligence tasks) are very close. They do not fully overlap, because they probably call for different combinations of the same processes or for slightly different additional processes, but nevertheless they all account for a large part of the total variance in the dependent variable and for most of the age-related variance. 

## 6. Discussion: A Proposition in Terms of Vicarious Processes

We have tried to convince the reader, throughout this paper, that the three traditions of research discussed in this issue, that is, psychometrics or intelligence research, cognitive psychology, and Piagetian or neo-Piagetian developmental psychology, are much closer than they appear [1,5,71]. Theoretically, they pursue the same objectives, while each has focused on one of the sources of variation of a same object of study: age differences, individual differences, or experimental variations. We have argued that all of these sources of variation should be integrated. As mentioned in the introduction, Piaget insisted long ago on the necessity to study the development of a structure to understand its functioning in its final state. This argument is even more relevant nowadays and can be applied to all sorts of cognitive functioning and not only to structures; it should be taken into consideration by both psychometricians and cognitive psychologists. The way a given structure has developed not only helps understanding it, but may also account for a substantial variation within and among young adults.

Several authors have argued that focusing on the mean performance of a group is not sufficient, not only because it does limit the generalizability, but most importantly because it may lead to wrong conclusions [72]. It is thus also necessary to focus on the individual and to take intra-individual variability into account [1,16,73,74,75]. Finally, wiping out the importance of variations in situations (experimental variations) leads to missing the existence of finer processes and neglecting the variability of human beings across behavioral measures, when such variability can serve, among others, as indicator of flexibility. 

Our argument is that a unified vision is possible, provided one is (a) interested in the underlying processes and not only in the variables; (b) willing to adopt a multidimensional view and (c) granting a fundamental role to individual differences. One way to reconcile the different approaches is to consider that several general processes are at work in all individuals and that they develop with age and allow them to deal with numerous types of situations. However, their relative weight and interactions might differ among individuals. Thus, Reuchlin, a French differentialist, suggested the concept of “*vicariances*” [76]. Vicarious processes are assumed to be present, to various degrees, in all individuals, and they are more or less necessary, depending on the situation. They differ by their evocability, that is by the ease with which they can be activated either as a function of the type of task or of the individual (see also [62,77]). 

In the complex tasks that were illustrated in the previous section, three general processes at least might be at work: the processing resources available to the individual such as WM capacity or processing capacity in some approaches or M-power in Pascual-Leone’s model, an inhibitory mechanism and a set of executive schemes. First, due to their complexity, these tasks require a relatively high processing capacity (several schemes and logical rules to be activated simultaneously) to be solved or to reach a given intermediate level. The capacity or processing resources required obviously varies across situations; it is usually relatively high in WM tasks or in Piagetian tasks, even just to reach an intermediate level. Note, in passing, that processing capacity and processing speed^4^, a mechanism often invoked in developmental models [79,80], may be considered as alternative definitions of the expression of a same mechanism: A larger processing capacity allows to process information faster; reciprocally greater speed of processing allows processing more information in a given time window. At the present time, it does not seem possible to dissociate speed from amount of resources or capacity at the behavioral level. Second, inhibition has often been invoked as a causal factor of development, or of individual differences [58,81,82]. Third, the main role of executive schemes is to allocate these resources. Each of these three broad mechanisms should not be considered as a single, alternative explanation, but they work together. There are most probably also other processes at work, but the change in these three general mechanisms with age and their variation in relative weight and interaction are probably sufficient to account not only for developmental differences but also for the enormous individual and behavioral variability observed in cognitive tasks. 

One could perhaps also argue that these three general mechanisms (processing capacity, inhibition, and executive schemes^5^) could be reduced to a single general process, such as a general factor. We would like to respond drawing upon several arguments, theoretical, methodological, and empirical. First, as mentioned above, a general factor does not rest on a single, general process (e.g., [25,85]). Second, the enormous individual variability that is observed, within a single age group, is often qualitative and not only quantitative. If there was only one underlying dimension, all individuals could be ranked; consequently, at least two dimensions or processes are necessary to allow (a) a dynamic interplay between them that can account for development, and (b) variations of such interplay across individuals (see, for instance, [77]). Third, in several structural equations modeling used on the data collected in our lifespan studies, we modeled the relationships between Age, Inhibition, Processing speed, WM, and their influence on behavior in a fluid intelligence task. In accord with our hypothesis, the best model was a cascade one, in which Inhibition and Processing speed accounted for age differences in WM tasks that in turn accounted for performance observed in a fluid intelligence task, or in other cognitive tasks. Comparing a group of children and young adults and a group of young and older adults, we found that these three mechanisms were at work in these two life periods, but in a different dosage. Also, and importantly for the present argument, using either a single general factor (the latent variables did indeed present a relatively strong correlation), reducing the three latent variables to two only, or changing somewhat the direction of influence, yielded a worse fit. Finally, although we agree that a relatively simple view should be adopted, we do not agree with the necessity to define a hundred or more underlying processes (see, for a review [86]); it should not be too simple either^6^. For instance, Reuchlin [17] argued that a structural approach (i.e., the study of the relationships between observed or latent variables) might be the opposite of the idea of parsimony or economy in psychology (Ockham’s razor or Morgan’s canon). Structural models admit several variables, relations, and most importantly allow for vicarious processes. According to Reuchlin, a criterion of parsimony is not the most pertinent for describing psychological functioning, and the “true” functioning of the knowledge system is not necessarily the simplest. 

In conclusion, we want to argue that most situations cannot be equated with a specific, single underlying process, as has been argued too often in cognitive psychology. This fractionation has led to an unnecessary multiplication of processes. Yet, a single, general process or mechanism, as factor analysts have sometimes believed, is not sufficient either. A hierarchical approach, in which few, general processes are assumed, that can perhaps be decomposed further into finer processes, should be able to account for most of the age and individual differences observed. This is to the condition that a model in terms of a dynamic interplay among vicarious processes is adopted: These few mechanisms are present at all ages, and in all individuals, but they may vary in terms of their relative weight. This weight can also change as a function of age, time (explaining for instance a number of learning effects observed) and situations. Thus, a differential reliance on such processes accounts for the large inter- and intraindividual variability observed in intelligence or psychometric research. Finally, a reconciliation between the three visions examined in this special issue requires adopting more complex, multivariate experimental designs that would make it possible to assess the effects of situations, age, and both inter and intraindividual differences in the same set of variables. 

## Footnotes

1 We are adding the perspective of cognitive psychology to those included in the title of this special issue, because most recent studies of cognitive development and of intelligence are referring to constructs developed within experimentalist approaches as exemplified by cognitive psychology.

2 Our argument, throughout this paper, is that intelligence is an object of study that has been addressed from different perspectives. However, in the Anglo-Saxon tradition, the term “intelligence research” has usually been associated with the psychometric approach. This is not the case in other research traditions, notably in Francophone ones (see below).

3 For instance, Anderson (2001) [13] suggested that changes with age were related with response selection rather than with speed of processing.

4 For Anderson ([13]; see also this issue), processing speed explains better individual differences than developmental differences. In contrast, processing capacity as conceived by neo-Piagetians is a major actor in development, and we contend that, behaviorally speaking, it is not possible to dissociate speed from the quantity of resources available. Also, in our lifespan studies, we observed that processing speed always accounts for a large part of the age differences in WM tasks [65,78].

5 Let us specify, in response to a referee’s question, that other terms can be, and are, used to refer to the same or similar mechanisms, and we could easily also use them. Two points seem crucial, however: (a) the necessity to dissociate tasks from underlying processes; and (b) think in terms of multidimensionality. Thus, talking of a WM mechanism or system is misleading; there are *WM tasks* that call for an underlying processing capacity (for short, sometime we used the term WM capacity), or attentional capacity, or M-power in Pascual-Leone’s terms; such a mechanism corresponds to the quantity of elements (items of information, or chunks) that can be simultaneously activated in a given mental operation. Behaviorally, it can be measured most closely by well-designed WM tasks (see also, for a recent discussion, [83]). Once again, a given task always calls for more than a single process. We would also like to argue that attentional control, another term frequently used, rests on all three mechanisms [84].

6 We would like to thank an anonymous referee who called our attention to a very nice quote attributed to Einstein: “Everything should be made as simple as possible, but not simpler”.

## References

[1] Case R., Demetriou A., Platsidou M., Kazi M. (2001). Integrating concepts and tests of intelligence from the differential and developmental traditions. Intelligence.

[2] Cronbach L.J. (1957). The two disciplines of scientific psychology. Am. Psychol..

[3] Resnick L.B., Resnick L.B. (1976). Introduction: Changing conceptions of intelligence. The Nature of Intelligence.

[4] Reuchlin M. (1964). L’intelligence: Conception génétique opératoire et conception factorielle. Rev. Suisse Psychol. Pure Appl..

[5] De Ribaupierre A., Pascual-Leone J. (1984). Pour une intégration des méthodes en psychologie: Approches expérimentale, psycho-génétique et différentielle. L’Année Psychol..

[6] Cattell R.B. (1952). The three basic factor-analytic research designs—Their interrelations and derivatives. Psychol. Bull..

[7] Cattell R.B., Nesselroade J.R., Cattell R.B. (1988). The data Box. Its ordering of total resources in terms of possible relational systems. Handbood of Multivariate Experimental Psychology.

[8] Binet A., Simon T. (1908). Le développement de l’intelligence chez les enfants. L’Année Psychol..

[9] Hunt E. (2011). Human Intelligence.

[10] Piaget J. (1936). La Naissance de L’intelligence Chez L'enfant.

[11] Piaget J. (1947). La Psychologie de L’intelligence.

[12] Lautrey J., de Ribaupierre A., Sternberg R.J. (2004). Psychology of human intelligence in France and French-speaking Switzerland. International Handbook of Intelligence.

[13] Anderson M. (2001). Annotation: Conceptions of Intelligence. J. Child Psychol. Psychiatry.

[14] Anderson M., Sternberg R.J., Pretz J.E. (2005). Marrying intelligence and cognition: A developmental view. Cognition and Intelligence: Identifying the Mechanisms of the Mind.

[15] Cornoldi C. (2006). The Contribution of Cognitive Psychology to the Study of Individual Cognitive Differences and Intelligence (Special Issue of the European Journal of Cognitive Psychology).

[16] Demetriou A., Spanoudis G., Shayer M. (2013). Developing intelligence: Is a comprehensive theory possible?. Intelligence.

[17] Reuchlin M. (1995). Totalités, Eléments, Structures en Psychologie.

[18] Reuchlin M. (1981). Apports de la méthode différentielle à la psychologie générale. J. Psychol..

[19] Lautrey J., de Ribaupierre A., Rieben L. (1985). Intra-individual variability in the development of concrete operations. Relations between logical and infra-logical operations. Genet. Soc. Gen. Psychol. Monogr..

[20] Longeot F. (1969). Psychologie Différentielle et Théorie Opératoire de L’intelligence.

[21] Rieben L., de Ribaupierre A., Lautrey J. (1990). Structural invariants and individual modes of processing: On the necessity of a minimally structuralist approach of development for education. Arch. Psychol..

[22] Larivée S., Normandeau S., Parent S. (2000). The French connection: Some contributions of French-language research in the post-Piagetian era. Child Dev..

[23] Horn J.L., Cattell R.B. (1966). Refinement and test of the theory of flluid and crystallised general intelligences. J. Educ. Psychol..

[24] Baltes P.B., Staudinger U.M., Lindenberger U. (1999). Lifespan psychology: Theory and application to intellectual functioning. Annu. Rev. Psychol..

[25] Van der Maas H., Dolan C.V., Grasman R.P.P., Wicherts J.M., Huizenga H.M., Raijmakers M.E.J. (2006). A Dynamical Model of General Intelligence: The Positive Manifold of Intelligence by Mutualism. Psychol. Rev..

[26] Carroll J.B. (1993). Human Cognitive Abilities.

[27] Mcgrew K. (2009). CHC theory and the Human Cognitive Abilities Project: Standing on the shoulders of the giants of psychometric intelligence research. Intelligence.

[28] Friedman N.P., Miyake A. (2004). The Relations Among Inhibition and Interference Control Functions: A Latent-Variable Analysis. J. Exp. Psychol. Gen..

[29] Miyake A., Friedman N.P. (2012). The Nature and Organization of Individual Differences in Executive Functions: Four General Conclusions. Curr. Dir. Psychol. Sci..

[30] Miyake A., Friedman N.P., Emerson M.J., Witzki A.H., Howerter A., Wager T.D. (2000). The unity and diversity of executive functions and their contributions to complex “frontal lobe” tasks: A latent variable analysis. Cogn. Psychol..

[31] Jensen A.R. (2012). The theory of intelligence and its measurement. Intelligence.

[32] Kranzler J.H., Jensen A.R. (1991). The nature of psychometric g: Unitary process or a number of independent processes. Intelligence.

[33] Miller L.T., Vernon P.A. (1992). The general factor in short-term memory, intelligence and reaction time. Intelligence.

[34] Vernon P.A. (1986). Inspection time: Does it measure intelligence?. Personal. Individ. Differ..

[35] Ackerman P.L., Beier M.E., Boyle M.O. (2002). Individual differences in working memory within a nomological network of cognitive and perceptual speed abilities. J. Exp. Psychol. Gen..

[36] Ackerman P.L., Beier M.E., Boyle M.O. (2005). Working Memory and Intelligence: The Same or Different Constructs?. Psychol. Bull..

[37] Kyllonen P., Christal R.E. (1990). Reasoning ability is (little more than) working-memory capacity?. Intelligence.

[38] Conway A.R., Kane M.J., Engle R.W. (2003). Working memory capacity and its relation to general intelligence. Trends Cogn. Sci..

[39] Engle R.W., Kane M.J., Tuholski S.W., Miyake A., Shah P. (1999). Individual differences in working memory capacity and what they tell us about controlled attention, general fluid intelligence and functions of the prefrontal cortex. Models of Working Memory: Mechanisms of Active Maintenance and Executive Control.

[40] Engle R.W., Tuholski S.W., Laughlin J.E., Conway A.R.A. (1999). Working memory, short-term memory, and general fluid Intelligence : A latent-variable approach. J. Exp. Psychol. Gen..

[41] Gignac G.E. (2014). Fluid intelligence shares closer to 60% of its variance with working memory capacity and is a better indicator of general intelligence. Intelligence.

[42] Gignac G.E., Watkins M.W. (2015). There may be nothing special about the association between working memory capacity and fluid intelligence. Intelligence.

[43] Shipstead Z., Harrison T.L., Engle R.W. (2016). Working Memory Capacity and Fluid Intelligence: Maintenance and Disengagement. Perspect. Psychol. Sci..

[44] Colom R., Rebollo I., Abad F.J., Shih P.C. (2006). Complex span tasks, simple span tasks, and cognitive abilities: A reanalysis of key studies. Mem. Cognit..

[45] Case R. (1985). Intellectual Development. Birth to Adulthood.

[46] Case R., Sternberg R.J., Berg C.A. (1992). Neo-piagetian theories of child development. Intellectual Development.

[47] Dasen P.R., de Ribaupierre A. (1987). Neo-piagetian theories: Cross-cultural and differential perspectives. Int. J. Psychol..

[48] De Ribaupierre A. (1997). Les modèles néo-piagétiens: Quoi de nouveau?. Psychol. Fr..

[49] Pascual-Leone J. (1970). A mathematical model for the transition rule in Piaget’s developmental stages. Acta Psychol..

[50] Pascual-Leone J., Goodman D.R. (1979). Intelligence and experience: A neo-Piagetian approach. Instr. Sci..

[51] Pascual-Leone J. (1987). Organismic processes for neo-Piagetian theories: A dialectical causal account of cognitive development. Int. J. Psychol..

[52] De Ribaupierre A., Bailleux C. (1994). Developmental change in a spatial task of attentional capacity: An essay toward an integration of two working memory models. Int. J. Behav. Dev..

[53] De Ribaupierre A., Bailleux C. (2000). The development of working memory: Further note on the comparability of two models of working memory. J. Exp. Child Psychol..

[54] Pascual-Leone J., Ijaz I., Samuda R.J., Kong S.L., Cummins J., Pascual-Leone J., Lewis J. (1989). Mental capacity testing as a form of intellectual-developmental assessment. Assessment and Placement of Minority Students.

[55] Pascual-Leone J. (2000). Reflections on working memory: Are the two models complementary?. J. Exp. Child Psychol..

[56] Baddeley A.D. (1986). Working Memory.

[57] Cowan N. (2005). Working Memory Capacity.

[58] Pascual-Leone J., Johnson J., Barrouillet P., Gaillard V. (2011). A developmental theory of mental attention: Its applications to measurement and task analysis. Cognitive Development and Working Memory: A Dialogue Between Neo-Piagetian and Cognitive Approaches.

[59] Case R. (1992). The Mind’s Staircase: Exploring the Conceptual Underpinnings of Children’s Thought and Knowledge.

[60] Fischer K.W. (1980). A theory of cognitive development: The control and construction of hierarchies of skills. Psychol. Rev..

[61] Fischer K.W., Silvern L. (1985). Stages and individual differences in cognitive development. Annu. Rev. Psychol..

[62] De Ribaupierre A., Case R., Edelstein W. (1993). Structural invariants and individual differences: On the difficulty of dissociating developmental and differential processes. The New Structuralism in Cognitive Development: Theory and Research on Individual Pathways.

[63] Pascual-Leone J., Morra S., Reese H.W. (1991). Horizontality of water level: A neopiagetian developmental review. Advances in Child Development and Behavior.

[64] Demetriou A., Spanoudis G., Shayer M., Mouyi A., Kazi S., Platsidou M. (2013). Cycles in speed-working memory-G relations: Towards a developmental—Differential theory of the mind. Intelligence.

[65] De Ribaupierre A., Fagot D., Lecerf T., Barrouillet P., Gaillard V. (2011). Working memory capacity and its role in cognitive development. Cognitive Development and Working Memory.

[66] De Ribaupierre A., Neirynck I., Spira A. (1989). Interactions between basic capacity and strategies in children’s memory: Construction of a developmental paradigm. Curr. Psychol. Cognit. (CPC).

[67] Inhelder B., Piaget J. (1955). De la Logique de L’enfant à la Logique de L’adolescent: Essai Sur la Construction des Structures Opératoires Formelles.

[68] Piaget J., Inhelder B. (1966). L’image Mentale Chez L’enfant.

[69] Piaget J., Inhelder B., Szeminska A. (1948). La Géométrie Spontanée Chez L’enfant.

[70] Salthouse T.A. (1992). Mechanisms of Age–Cognition Relations in Adulthood.

[71] Pascual-Leone J. (1969). Cognitive Development and Cognitive Style: A General Psychological Integration.

[72] Nesselroade J.R., Downs R.M., Liben L.S., Palermo D.S. (1991). The warp and woof of the developmental fabric. Views of Development, the Environment, and Aesthetics: The Legacy of Joachim F. Wohlwill.

[73] Mella N., Fagot D., de Ribaupierre A. (2016). Dispersion in cognitive functioning: Age differences over the lifespan. J. Clin. Exp. Neuropsychol..

[74] De Ribaupierre A., Juhel J., Rouxel G. (2015). Pourquoi faut-il étudier la variabilité intra-individuelle lorsqu’on s’intéresse au développement cognitif?. Différences et Variabilité en Psychologie.

[75] De Ribaupierre A. (2015). Why Should Cognitive Developmental Psychology Remember that Individuals Are Different?. Res. Hum. Dev..

[76] Reuchlin M. (1978). Processus vicariants et différences individuelles. J. Psychol..

[77] Lautrey J., Sternberg R.J., Lautrey J., Lubart T. (2003). A Pluralistic Approach to Cognitive Differentiation and Development. Models of Intelligence: International Perspectives.

[78] De Ribaupierre A. (2000). Working Memory and Attentional Processes across the Lifespan.

[79] Coyle T.R., Pillow D.R., Snyder A.C., Kochunov P. (2011). Processing Speed Mediates the Development of General Intelligence (*g*) in Adolescence. Psychol. Sci..

[80] Kail R., Salthouse T. (1994). Processing speed as a mental capacity. Acta Psychol..

[81] Dempster F.N. (1991). Inhibitory processes: A neglected dimension of intelligence. Intelligence.

[82] Engle R.W., Conway A.R.A., Tuholski S.W., Shisler R.J. (1995). A Resource Account of Inhibition. Psychol. Sci..

[83] Arsalidou M., Im-Bolter N. (2016). Why parametric measures are critical for understanding typical and atypical cognitive development. Brain Imaging Behav..

[84] De Ribaupierre A., Perrig W., Grob A. (2000). Working memory and attentional control. Control of Human Behavior, Mental Processes, and Consciousness.

[85] Van der Maas H., Kan K.-J., Borsboom D. (2014). Intelligence Is What the Intelligence Test Measures. Seriously. J. Intell..

[86] Salthouse T.A. (1991). Theoretical Perspectives on Cognitive Aging.

